# Beyond dichotomy: patterns and amplitudes of SSEPs and neurological outcomes after cardiac arrest

**DOI:** 10.1186/s13054-019-2510-x

**Published:** 2019-06-18

**Authors:** Sang Hoon Oh, Kyu Nam Park, Seung Pill Choi, Joo Suk Oh, Han Joon Kim, Chun Song Youn, Soo Hyun Kim, Kiyuk Chang, Seong Hoon Kim

**Affiliations:** 1 Department of Emergency Medicine, Seoul St. Mary’s Hospital, College of Medicine, The Catholic University of Korea, 222 Banpo-daero, Seocho-gu, Seoul, 06591 Republic of Korea; 2 Department of Emergency Medicine, Yeouido St. Mary’s Hospital, College of Medicine, The Catholic University of Korea, Seoul, Republic of Korea; 3 Department of Emergency Medicine, Uijeongbu St. Mary’s Hospital, College of Medicine, The Catholic University of Korea, Seoul, Republic of Korea; 4 Division of Cardiology, Department of Internal Medicine, Seoul St. Mary’s Hospital, College of Medicine, The Catholic University of Korea, Seoul, Republic of Korea; 5 Department of Neurology, Uijeongbu St. Mary’s Hospital, College of Medicine, The Catholic University of Korea, Seoul, Republic of Korea

**Keywords:** Heart arrest, Induced hypothermia, Evoked potentials, Prognosis

## Abstract

**Background:**

We hypothesized that the absence of P25 and the N20–P25 amplitude in somatosensory evoked potentials (SSEPs) have higher sensitivity than the absence of N20 for poor neurological outcomes, and we evaluated the ability of SSEPs to predict long-term outcomes using pattern and amplitude analyses.

**Methods:**

Using prospectively collected therapeutic hypothermia registry data, we evaluated whether cortical SSEPs contained a negative or positive short-latency wave (N20 or P25). The N20–P25 amplitude was defined as the largest difference in amplitude between the N20 and P25 peaks. A good or poor outcome was defined as a Glasgow-Pittsburgh Cerebral Performance Category (CPC) score of 1–2 or 3–5, respectively, 6 months after cardiac arrest.

**Results:**

A total of 192 SSEP recordings were included. In all patients with a good outcome (*n* = 51), both N20 and P25 were present. Compared to the absence of N20, the absence of N20–P25 component improved the sensitivity for predicting a poor outcome from 30.5% (95% confidence interval [CI], 23.0–38.8%) to 71.6% (95% CI, 63.4–78.9%), while maintaining a specificity of 100% (93.0–100.0%). Using an amplitude < 0.64 μV, i.e., the lowest N20–P25 amplitude in the good outcome group, as the threshold, the sensitivity for predicting a poor neurological outcome was 74.5% (95% CI, 66.5–81.4%). Using the highest N20–P25 amplitude in the CPC 4 group (2.31 μV) as the threshold for predicting a good outcome, the sensitivity and specificity were 52.9% (95% CI, 38.5–67.1%) and 96.5% (95% CI, 91.9–98.8%), respectively. The predictive performance of the N20–P25 amplitude was good, with an area under the receiver operating characteristic curve (AUC) of 0.94 (95% CI, 0.90–0.97). The absence of N20 was statistically inferior regarding outcome prediction (*p* < 0.05), and amplitude analysis yielded significantly higher AUC values than did the pattern analysis (*p* < 0.05).

**Conclusions:**

The simple pattern analysis of whether the N20–P25 component was present had a sensitivity comparable to that of the N20–P25 amplitude for predicting a poor outcome. Amplitude analysis was also capable of predicting a good outcome.

**Electronic supplementary material:**

The online version of this article (10.1186/s13054-019-2510-x) contains supplementary material, which is available to authorized users.

## Background

Reliable early prognostication of neurological recovery in comatose cardiac arrest survivors receiving therapeutic hypothermia (TH) remains a major clinical challenge [1]. Sedation with or without paralysis during TH may delay neurological recovery [2]. Therefore, these confounders might limit the value of a neurological examination and necessitate other multimodal prognostic approaches.

Of these modalities, somatosensory evoked potential (SSEP) measurement is a noninvasive bedside technique that can even be used for unstable critically ill patients and is less confounded by sedation or hypothermia than electroencephalography (EEG). The bilateral absence of N20 on days 1–3 or later after the return of spontaneous circulation (ROSC) is currently the most reliable predictor of a poor outcome, with a false-positive rate (FPR) close to 0% [3–7]. However, the current interpretation of SSEPs has several limitations. As the N20 interpretation is dichotomous (absent/present), discrimination between a low-amplitude and absent N20 is crucial. High noise levels and artifacts may affect the ability to discern a low-amplitude N20 and impede reliable interpretation [8]. These problems could also produce interobserver disagreement and false-positive cases. Furthermore, the absence of N20 has a low sensitivity for predicting a poor outcome [9–12]. A few studies have suggested 0.1–0.3 μV as a reliable noise level for cortical SSEPs [8, 13–15]. Several features have recently been studied for increasing the sensitivity of SSEPs [16]. Endisch et al. performed a large prospective study on the relationship between cortical SSEP amplitudes and short-term neurological outcomes and found that absent or very low-amplitude SSEPs appear to be highly predictive of a poor outcome, with high sensitivity [17]. Kim et al. reported that the absence of P25/30 has better sensitivity than the absence of N20 for predicting a poor neurological outcome at hospital discharge [18].

However, neither of these recent analyses has been validated, especially in patients who were followed for a long period. We hypothesized that the absence of P25 and the amplitude of the N20–P25 complex have higher sensitivity than the absence of N20 for predicting a poor neurological outcome after 6 months. The aims of this study were to evaluate and compare the predictive values of SSEPs by using pattern and amplitude analyses of cortical short-latency SSEPs.

## Methods

### Study design and patients

This retrospective observational study used prospectively collected data from the TH registry at Seoul St. Mary’s Hospital in Seoul, Korea, between February 2009 and May 2017. We included patients who were older than 18 years, were treated with TH after cardiac arrest, and had a SSEP measurement. Unsuitable SSEP recordings were excluded. Our institutional ethics committee approved this study, and the requirement for consent was waived because of the retrospective nature of the study.

### Postcardiac arrest care

During the study period, all patients who were comatose after ROSC were considered eligible for TH at 33 °C for 24 h. After the target temperature of 33 °C was maintained for 24 h, controlled rewarming at a rate of 0.25 °C/h was performed until the temperature reached 36.5 °C. Patients received sedatives and neuromuscular blockers via the combination of midazolam and rocuronium during the induction period; this treatment was reduced during rewarming and discontinued as soon as the central temperature reached 35 °C.

A standardized approach for making a prognosis was applied for all patients. Comatose patients who were successfully resuscitated underwent brain computed tomography immediately. All patients were monitored via amplitude-integrated EEG using a combined single- or multichannel device simultaneously with the initiation of TH. Measurements of serum biomarkers, including neuron-specific enolase (NSE), were obtained immediately after ROSC and repeated 24, 48, and 72 h later. Neurological examinations were performed by emergency physicians and by intensive care unit nurses at other time points according to standard practices. The SSEP measurements and diffusion-weighted imaging (DWI) were usually performed after rewarming. The prognosis was made using a combination of predictors of a poor outcome to improve the prognosis in the setting of TH.

Because withdrawal of life-sustaining treatment (WLST) is legally prohibited in South Korea, it was not considered for any participant according to the prognosis. However, do-not-resuscitate (DNR) orders are legal and socially acceptable. Therefore, if the family did not want further resuscitation to be performed after the prognosis was made, it was not performed.

### SSEP recordings

SSEPs were recorded routinely as an outcome predictor after the completion of rewarming. However, because SSEPs could not be recorded at night or over the weekend for practical reasons, the recording was initiated during TH in these cases. If a patient recovered consciousness prior to the SSEPs being recorded, the SSEP recording was not obtained.

SSEPs were recorded using a Viking EDX (Natus Medical Incorporated, Pleasanton, CA, USA) by one technician with 20 years of experience. The median nerves were stimulated by surface electrodes at both wrists with the stimulus intensity necessary to evoke a clearly visible muscle twitch causing abduction of the thumb. The stimulus pulse duration was 0.2 ms, and the stimulus rate was 3.1 Hz. SSEP recordings were obtained from needle electrodes placed on the surface of the supraclavicular fossa, C2 spine, and C3’ or C4’ in accordance with the 10–20 international system, and the impedance was kept below 5 kΩ. The reference electrode was placed on the midfrontal area (Fz). The poststimulus recording time was 60 ms, and the bandwidth was 30–3000 Hz. A minimum of 300 stimulations were averaged per recording. In our recording protocol, it was not essential to include or superimpose at least two repeated recordings. When a recording had a high level of noise, we performed several additional tests to minimize the noise.

### Interpretation and analyses of SSEPs

All SSEP recordings were reinterpreted by the authors (JSO and SHK) from other institutions, who were blinded to the patient outcomes. When normal potentials over Erb’s point (N9) and the cervical spinal cord (N13) were present, the SSEPs were analyzed. By visually assessing all cortical recordings, we excluded those with a peak-to-peak noise amplitude > 0.25 μV after averaging [13, 17]. We defined N20 and P25 as negative waves at least 4.5 ms after the spinal peak, and the first positive wave was followed by N20 with a variable latency of 23–35 ms.

**Fig. 1:**
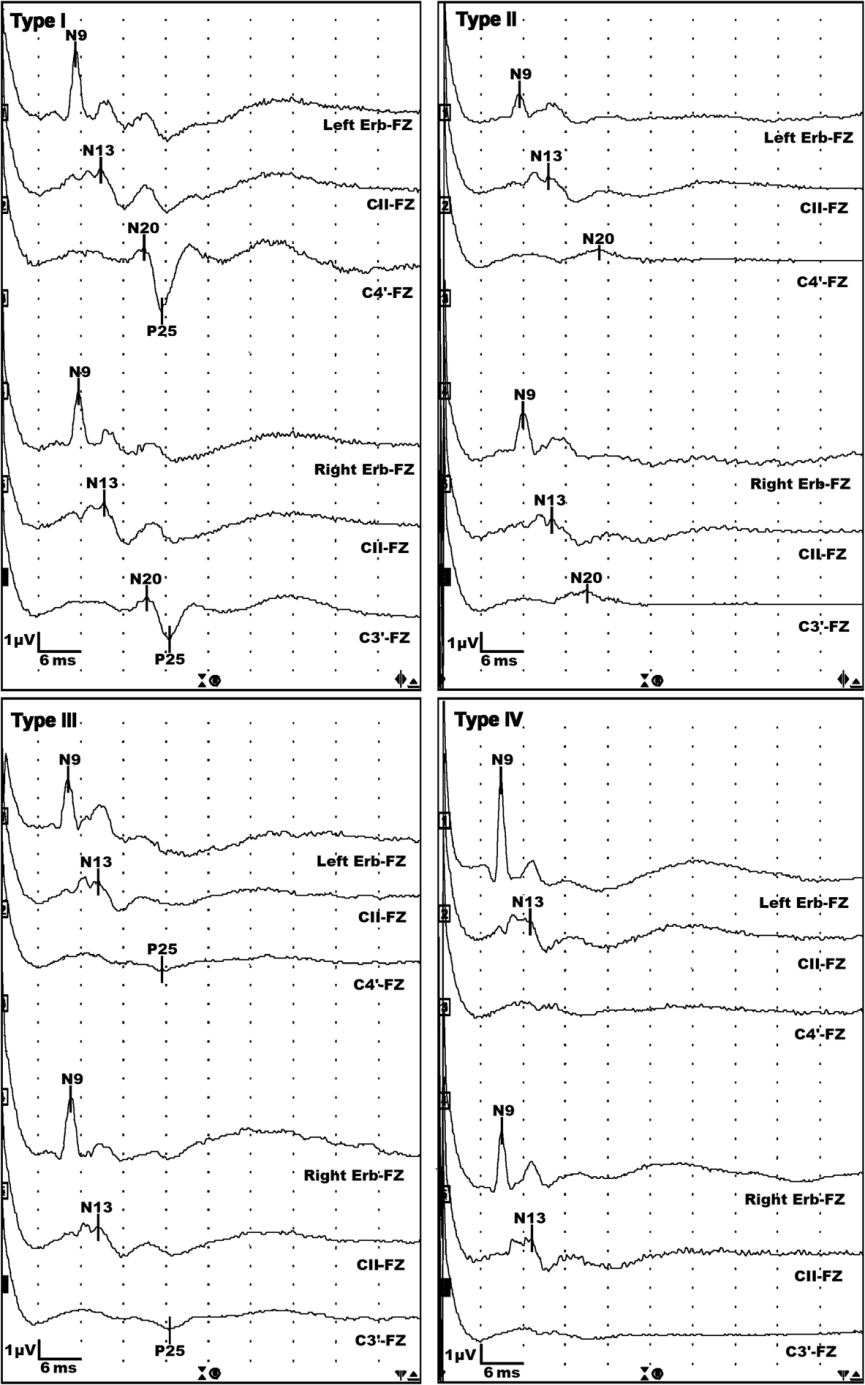
Pattern categories according to the presence of N20 or P25 on cortical somatosensory evoked potential recordings. Type I (*n* = 91): 51 cases of CPC 1–2 and 40 cases of CPC 3–5; type II (*n* = 58): all CPC 3–5; type III (*n* = 2): all CPC 4–5; and type IV (*n* = 41): all CPC 4–5. Erb, Erb’s point; FZ, frontal pole electrode; CII, C2 spinous process; C3’ and C4’, contralateral somatosensory cortexes; CPC, Cerebral Performance Category

The N20 and P25 peaks were considered absent if the amplitude was lower than 0.1 μV. The amplitude of the N20–P25 complex was determined as the difference between the amplitudes of the N20 and P25 peaks. If there was only N20 or P25, we defined the N20 or P25 amplitude as the N20–P25 amplitude. For pattern analysis, N20 or P25 was defined as absent if the specific wave was absent on both sides. According to whether N20 or P25 was absent, SSEP patterns were classified as type I for N20 (+)/P25 (+), type II for N20 (+)/P25 (−), type III for N20 (−)/P25 (+), and type IV for N20 (−)/P25 (−) (Fig. 1). To analyze the SSEP amplitude, we used the higher N20–P25 amplitude on both sides.

### Analyses of other outcome predictors

DWI findings were categorized into four patterns on the basis of the diffusion-restriction (hyperintense on DWI and low apparent diffusion coefficient values) lesions of the brain by a radiologist blind to the clinical outcome: (1) no diffusion-restriction lesion; (2) diffusion-restriction lesions, isolated cerebral cortex or deep gray matter; (3) multifocal lesions of diffusion-restriction, including both cerebral cortices and deep gray matter; and (4) global diffusion-restriction lesions in the brain [7, 19–21]. Representative images are presented in Additional file 1: Figure S1. To evaluate the prognostic performance of NSE, the highest serum level of NSE 48 and 72 h after ROSC was used (Roche Diagnostic, Mannheim, Germany). If the serum showed significant hemolysis, the NSE results were discarded.

### Outcome measurement

Finally, the neurological outcome at 6 months after ROSC was evaluated by the authors via a telephone interview. A good neurological outcome was defined as a Glasgow-Pittsburgh Cerebral Performance Category (CPC) score of 1 or 2, and a poor neurological outcome was defined as a CPC score of 3–5.

### Statistical analysis

The categorical variables are expressed as the number and percentage, and the continuous variables are expressed as the mean and standard deviation or the median and interquartile range (IQR) for a normal distribution. The performance of the outcome predictors was evaluated based on their sensitivity and specificity using an exact binomial 95% confidence interval (CI), and receiver operating characteristic (ROC) curve analysis was performed. Pairwise area under the ROC curve (AUC) comparisons were performed between various SSEP analyses using the nonparametric approach developed by DeLong et al. [22]. We also created combined models using logistic regression models. Bivariate associations between SSEP amplitudes and serum NSE levels were evaluated using the Pearson correlation coefficient. All statistical analyses were performed using IBM SPSS version 24 software (IBM, Armonk, NY, USA) and the MedCalc program (MedCalc Software, Mariakerke, Belgium). All *p* values were two-tailed, and *p* < 0.05 was considered significant.

## Results

### Baseline characteristics of participants

**Fig. 2:**
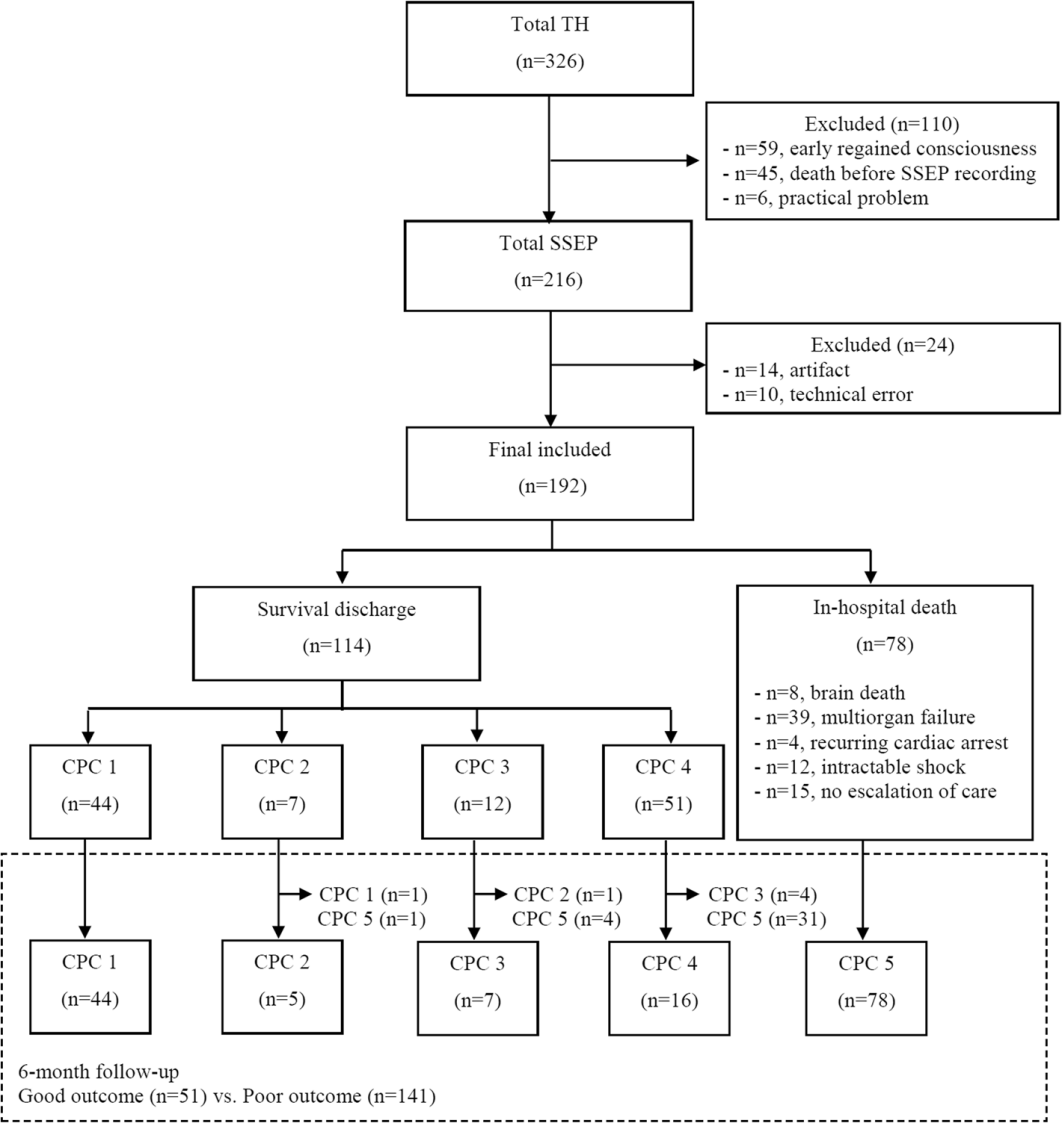
Flow chart for inclusion of the study patients. TH, therapeutic hypothermia; SSEP, somatosensory evoked potential

Over the study period, the data for 326 adult cardiac arrest patients treated with TH were entered into the registry. SSEPs were not recorded for 110 of these patients because they regained consciousness immediately after rewarming (*n* = 59), death occurred before SSEPs were recorded (*n* = 45), or practical considerations prevented SSEPs from being recorded (*n* = 6). Of the 216 recordings, 24 recordings were excluded from this study because of unsuitable results (14, artifact; 10, technical error). Ultimately, 192 patients were included in the analysis (Fig. 2). The baseline characteristics of the included patients are shown in Table 1. Of the included patients, 130 (67.7%) were male, and the mean patient age was 54.3 ± 16.3 years. A majority of the patients (172, 89.6%) had experienced an out-of-hospital cardiac arrest; 58 patients (30.2%) exhibited an initial shockable rhythm, and the mean time from cardiac arrest to ROSC was 33.5 ± 21.9 min. The median interval from ROSC to the SSEP recording was 41.6 h (IQR, 22.6–70.6 h). Seventy-eight patients died before hospital discharge, and their modes of death are presented in Fig. 2. At 6 months after ROSC, 51 (26.6%) patients exhibited a good neurological outcome and 141 patients (73.4%) exhibited a poor neurological outcome.

**Table 1:** Baseline characteristics of participants

Variables	Total participants (*n* = 192)
Male, *n* (%)	130 (67.7)
Age, years, mean ± SD	54.3 ± 16.3
OHCA, *n* (%)	172 (89.6)
Cardiac cause, *n* (%)	119 (62.0)
Witnessed, *n* (%)	136 (70.8)
Bystander CPR, *n* (%)	103 (53.6)
Shockable rhythm, *n* (%)	58 (30.2)
Time form arrest to ROSC, min, mean ± SD	33.5 ± 21.9
Time from ROSC to SSEP, h, median (IQR)	41.6 (22.6–70.6)
Length of hospital stays, days, median (IQR)	12.0 (6.0–23.0)
Neurological outcome 6 months after ROSC	
CPC 1, *n* (%)	45 (23.4)
CPC 2, *n* (%)	6 (3.1)
CPC 3, *n* (%)	11 (5.7)
CPC 4, *n* (%)	16 (8.3)
CPC 5, *n* (%)	114 (59.4)

*OHCA* out-of-hospital cardiac arrest, *CPR* cardiopulmonary resuscitation, *ROSC* return of spontaneous circulation, *SD* standard deviation, *SSEP* somatosensory evoked potential, *IQR* interquartile range, *CPC* Cerebral Performance Category

### Pattern analysis of SSEPs

Pattern analysis indicated that 91 recordings had both N20 and P25 and were considered SSEP type I. For approximately half of these recordings (56.0%, 51/91), the patient exhibited a good neurological outcome. The sensitivity of the type I pattern for predicting a good outcome was 100.0% (95% CI, 93.0–100.0%), with a specificity of 71.6% (95% CI, 63.4–78.9%). Patients with the type II pattern (*n* = 58) exhibited a poor neurological outcome (CPC 3, 3; CPC 4, 5; and CPC 5, 50). Type III and IV SSEP patterns were found for 2 and 41 recordings, respectively. All these patients exhibited a CPC score of 4 (*n* = 5) or 5 (*n* = 38).

The absence of N20 (types III and IV) and P25 (types II and IV) showed a sensitivity of 30.5% (95% CI, 23.0–38.8%) and 70.2% (95% CI, 61.9–77.6%), respectively, for predicting a poor outcome (Table 2). On the other hand, the sensitivity of the absence of N20 or P25 (types II, III, and IV) for predicting a poor outcome was 71.6% (95% CI, 63.4–78.9%).

**Table 2:** Sensitivity and specificity of somatosensory evoked potentials and other outcome predictors for 6-month neurological outcome

	Good outcome (*n* = 51)	Poor outcome (*n* = 141)	Poor outcome prediction		Good outcome prediction	
			Sensitivity % (95% CI)	Specificity % (95% CI)	Sensitivity % (95% CI)	Specificity % (95% CI)
N20–P25 pattern						
N20 (−), *n* (%)	0 (0.0)	43 (30.5)	30.5 (23.0–38.8)	100 (93.0–100.0)		
P25 (−), *n* (%)	0 (0.0)	99 (70.2)	70.2 (61.9–77.6)	100 (93.0–100.0)		
N20 (−) or P25 (−), *n* (%)	0 (0.0)	101 (71.6)	71.6 (63.4–78.9).	100.0 (93.0–100.0)		
N20–P25 amplitude						
< 0.64 μV, *n* (%)	0 (0.0)	105 (74.5)	74.5 (66.5–81.4)	100 (93.0–100.0)		
> 2.31 μV, *n* (%)	27 (52.9)	5 (3.5)			52.9 (38.5–67.1)	96.5 (91.9–98.8)
> 5.04 μV, *n* (%)	5 (9.8)	0 (0.0)			9.8 (3.3–21.4)	100.0 (97.4–100.0)
Peak level of NSE (*n* = 160)	(*n* = 48)	(*n* = 112)				
> 41.7 ng/mL, *n* (%)	4 (8.3)	91 (81.3)	81.3 (72.8–88.0)	91.7 (80.0–97.7)		
> 68.49 ng/mL, *n* (%)	0 (0.0)	68 (60.7)	60.7 (51.0–69.8)	100 (92.6–100.0)		
DWI lesion (*n* = 134)	(*n* = 36)	(*n* = 98)				
No diffusion-restriction lesion, *n* (%)	26 (72.2)	5 (5.1)			72.2 (54.8–85.8)	94.9 (88.5–98.3)
No lesion or isolated cortex or deep gray matter lesion, *n* (%)	34 (94.4)	8 (8.2)			94.4 (81.3–99.3)	91.8 (84.6–96.4)
Multifocal or global lesion, *n* (%)	2 (5.6)	90 (91.8)	91.8 (84.6–96.4)	94.4 (81.3–99.3)		

*CI* confidence interval, *NSE* neuron-specific enolase, *DWI* diffusion-weighted imaging

### Amplitude analysis of SSEPs

**Fig. 3:**
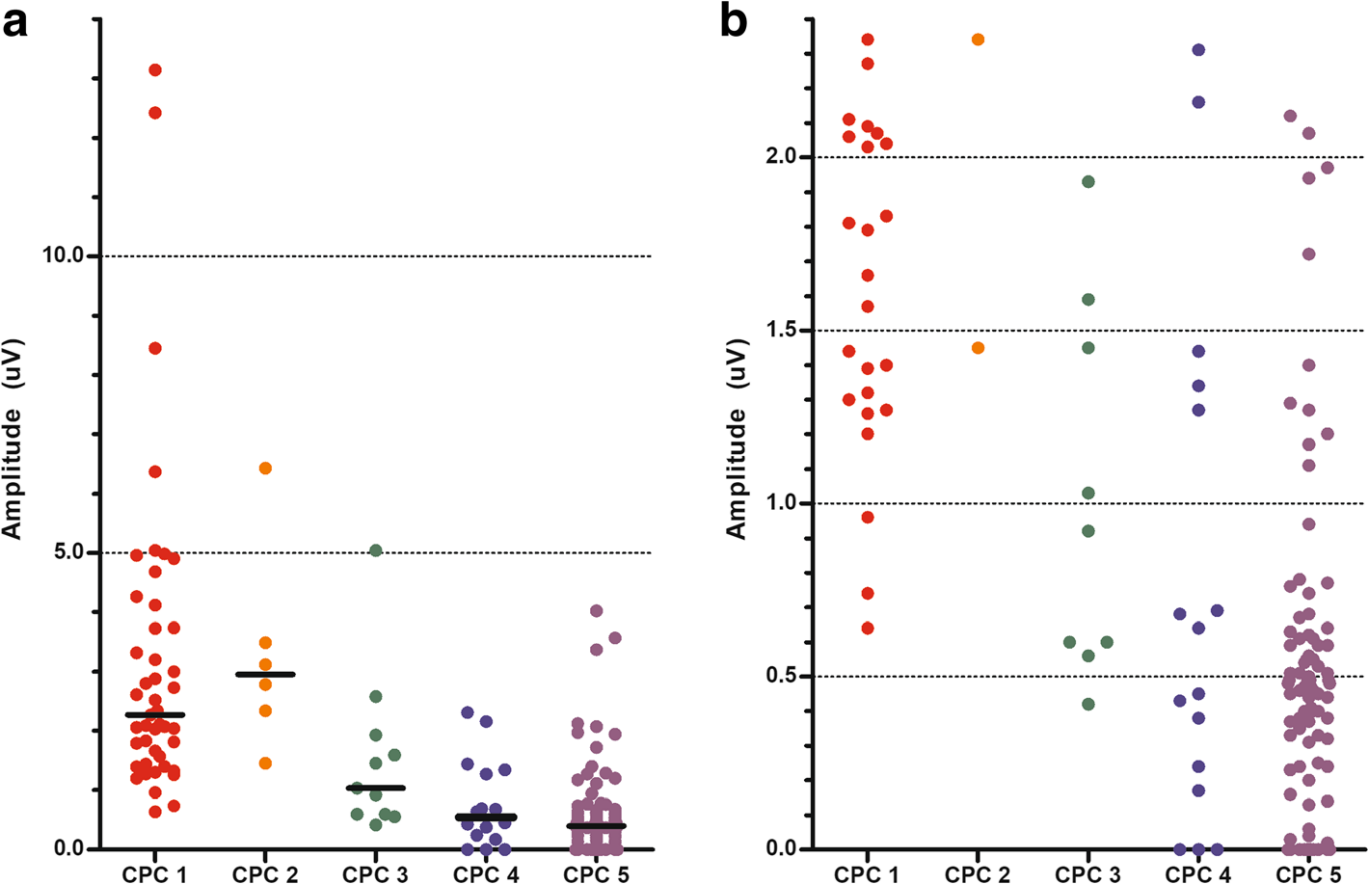
**a** Amplitudes of the N20–P25 component according to Cerebral Performance Category (CPC). Two patients in the CPC 3 group and 3 patients in the CPC 5 group had an amplitude above the upper limit for CPC 4 (> 2.31 μV). **b** *Y*-axis restricted to low amplitudes. The lower limit for CPC 1 and 2 was N20–P25 amplitudes > 0.64 μV

The N20–P25 amplitudes according to the CPC scores are shown in Fig. 3. The lowest N20–P25 amplitude in the good outcome group was 0.64 μV. Using an amplitude < 0.64 μV as the threshold, the sensitivity for predicting a poor neurological outcome was 74.5% (95% CI, 66.5–81.4%), with a 0% FPR (Table 2). Of the 11 recordings corresponding to a CPC score of 3, 4 had amplitudes lower than 0.64 μV (two of 0.60 and one each of 0.56 and 0.42 μV). Three of the corresponding patients had a CPC score of 4 at hospital discharge, while one patient showed a constant neurological status of CPC 3 for 6 months.

The highest N20–P25 amplitude in the poor outcome group was 5.04 μV (Fig. 3). The sensitivity of an N20–P25 amplitude > 5.04 μV as a threshold for predicting a good neurological outcome was 9.8% (95% CI, 3.3–21.4%), with a 0% FPR (Table 2). None of the 16 recordings of patients with a CPC score of 4 had an amplitude above 2.31 μV. On the other hand, only 5 recordings in the poor outcome group had an amplitude above 2.31 μV (CPC 3, 5.04 μV and 2.58 μV; CPC 5, 3.37, 3.57, and 4.02 μV). Using an amplitude > 2.31 μV, the sensitivity and specificity for predicting a good neurological outcome were 52.9% (95% CI, 38.5–67.1%) and 96.5% (95% CI, 91.9–98.8%), respectively.

**Fig. 4:**
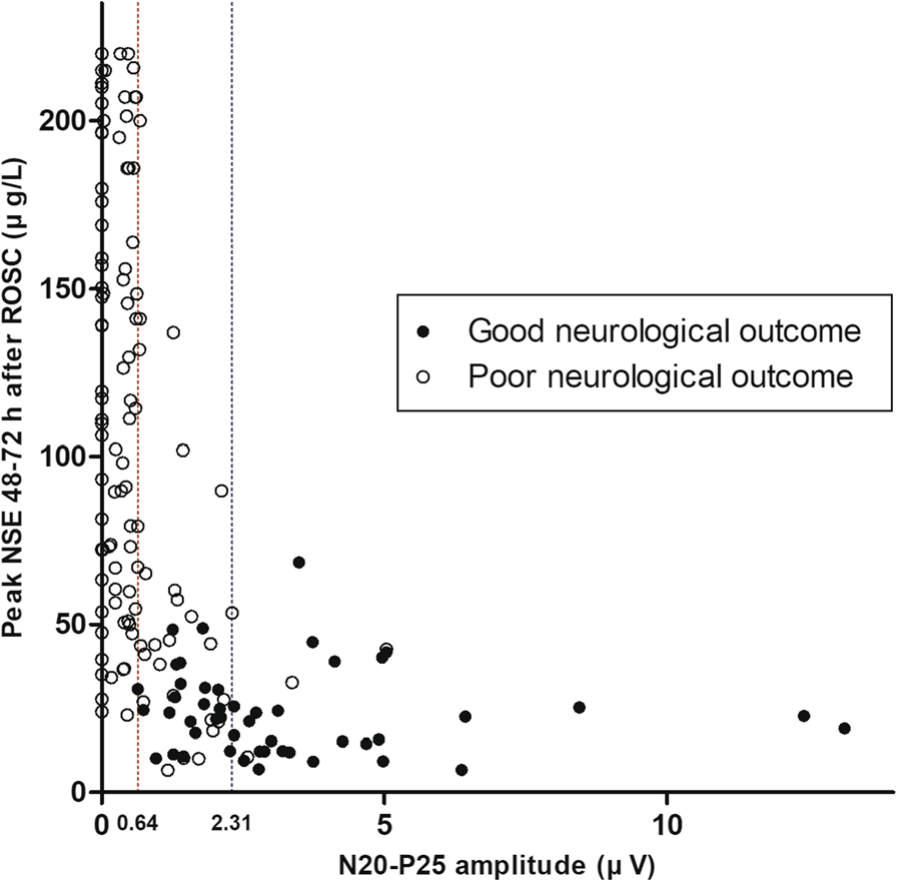
Scatter plots illustrating the associations between the peak levels of neuron-specific enolase between 48 and 72 h after the return of spontaneous circulation and the cortical amplitudes (*n* = 160)

Figure 4 displays scatter plots illustrating the associations between the peak serum levels of NSE at 48 and 72 h after ROSC and the cortical amplitudes in 160 recordings. The N20–P25 amplitude had a moderate negative correlation with the peak serum NSE level (*r* = − 0.470, *p* < 0.001).

### Comparisons between various SSEP analyses and other predictors

**Fig. 5:**
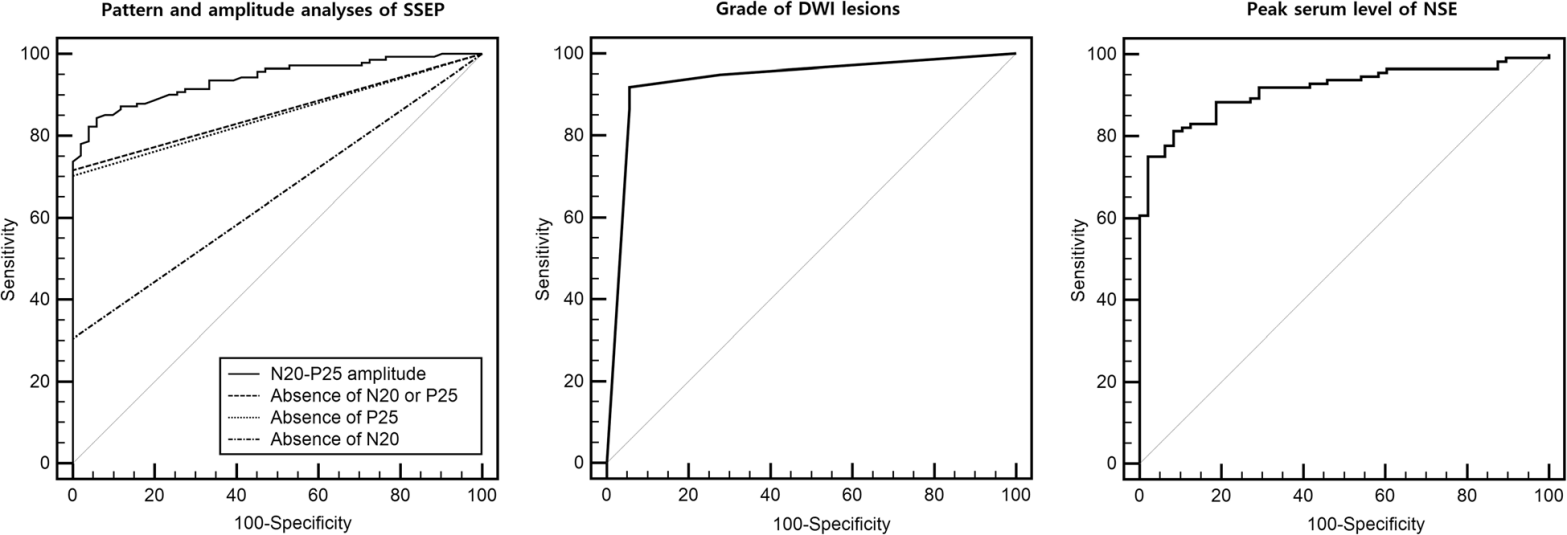
The receiver operating characteristic curves for Cerebral Performance Category scale scores 3–5 at 6 months showing the predictive powers of various SSEP analyses, the pattern of DWI, and the highest serum level of NSE. SSEP, somatosensory evoked potential; DWI, diffusion-weighted imaging; NSE, neuron-specific enolase

ROC analysis revealed that the absence of N20, the absence of P25, and the absence of N20 or P25 had an AUC of 0.65 (95% CI, 0.58–0.72), 0.85 (95% CI, 0.79–0.90), and 0.86 (95% CI, 0.80–0.90), respectively (Fig. 5). In the amplitude analysis, the predictive performance of the N20–P25 amplitude was good, with an AUC of 0.94 (95% CI, 0.90–0.97). The absence of N20 was statistically inferior for outcome prediction (*p* < 0.05). The amplitude analysis had a significantly higher AUC than did the pattern analysis (*p* < 0.05). On the other hand, the DWI pattern (*n* = 134) and the peak serum level of NSE (*n* = 160) had AUC values of 0.94 (95% CI, 0.88–0.97) and 0.91 (95% CI, 0.86–0.95), respectively (Fig. 5). The cutoff values and performances for each predictor are presented in Table 2.

In the subgroup that had all 3 prognostication tests performed (*n* = 114), the AUCs of the logistic regression models with combinations of SSEP results added to DWI and NSE results were calculated (Additional file 2: Figure S2). The AUCs increased when the absence of P25 or N20–P25 amplitude was added (AUC 0.95 and AUC 0.97, respectively), and the differences were statistically significant (Additional file 3: Table S1).

## Discussion

In this retrospective, single-center registry-based study, when we focused on the P25 peak or N20–P25 component, the sensitivity for predicting a poor outcome and the specificity for predicting a good outcome simultaneously increased more than twofold compared to those of the traditionally used pattern (absence of N20). On the other hand, the amplitude analysis was suitable for detailed prediction. An N20–P25 amplitude < 0.64 μV was a good predictor of a poor outcome, with prognostic value similar to that of the pattern analysis, while an amplitude > 2.31 μV was more capable of predicting a good outcome, with high specificity.

SSEP interpretations are traditionally dichotomous, with the absence or presence of N20 predicting a poor outcome or not. In our results, the absence of N20 or P25 guaranteed a poor long-term neurological outcome. However, the sensitivity for predicting a poor outcome was different between the two waves. This result is in line with the finding of a study by Kim et al. [18]. Although there was an insufficient number of type III SSEPs, all patients who experienced a good outcome showed the presence of both N20 and P25. The difference in prognostic value between the absence of N20 and P25 originated from the higher incidence of N20 than P25 in the poor outcome group (69.5% and 29.8%, respectively). Bauer et al. have reported that the extent of hypoxic-ischemic brain damage in cardiac arrest survivors increases along the afferent sensory pathway, with pronounced vulnerability of the thalamic and cortical brain regions [3]. Cortical N20 and P25 peaks were preserved in all patients with a CPC score of 1–2, whereas a stepwise decrease in the N20 and P25 cortical peaks was detectable in 63% and 59% of patients with a poor outcome, respectively. In a study by Kim et al., a significant decrease in detectable N20 and P25 peaks was observed in 30.9% and 11.1% of patients with a poor outcome, respectively [18].

However, the reason why the absence of P25 is more common than the absence of N20 in patients with a poor outcome is unknown. Both the N20 and P25 peaks are now widely accepted to be generated in the posterior bank of the central sulcus, corresponding to Brodmann area 3b [23–30]. However, it is possible that a minor contribution to the P25 component arises from the anterior bank of the central sulcus in Brodmann area 4 [29–31]. The issue of an additional contribution to changes in the P25 component by an anterior source in area 4 or by an additional radial source residing in area 1 may be clarified further only by applying multidipole localization algorithms to multichannel recordings [29–31].

The findings of the amplitude analysis are consistent with those of previous reports in the literature [17, 32, 33]. Various pathophysiological processes may decrease amplitudes in the acute phase of brain ischemia [34, 35]. However, amplitude assessment has largely been ignored in most clinical studies because the absolute value of the N20 amplitude varies by recording location and due to individual variability [36]. Some investigators have indicated that in the acute phase of stroke, the absolute N20 amplitude has significant predictive value regarding long-term functional recovery [37]. For comatose cardiac arrest patients, the N20 and N35 amplitudes were associated with functional outcomes at the time of discharge [32]. Among 49 anoxic coma patients, none with a baseline-to-peak N20 amplitude < 0.6 μV or a peak-to-peak short-latency wave amplitude < 1.2 μV recovered consciousness [33].

Our results for the amplitude threshold of short-latency waves were similar to those reported by Endisch et al. [17]. However, several differences in methodology deserve further mention. In our study, WLST, which could lead to an elevated low-amplitude threshold in patients with a good outcome, was not considered. Furthermore, the predictability of the SSEP amplitude might be overestimated in analyses considering short-term outcomes. We used the CPC score at 6 months after ROSC as the outcome and classified CPC 3 as the threshold for a poor outcome. Four patients with a CPC 4 at hospital discharge improved their neurological outcomes to CPC 3 after 6 months, and their lowest amplitude was 0.42 μV; two of them had type I SSEPs, and the rest had type II SSEPs. This result reveals that the lower amplitude threshold for a good outcome could be elevated after long-term follow-up. Although one patient in our cohort showed an improvement in neurological status from a CPC score of 3 to a CPC score of 2 after 6 months, the amplitude in his case was 2.34 μV, which was higher than the lowest amplitude in a survivor with a good outcome (0.62 μV) reported by Endisch et al. [17]. Interestingly, despite these differences, our absolute threshold value for the N20–P25 amplitude for predicting a poor outcome was comparable to the findings reported by Endisch et al. [17].

When we assumed that amplitudes above 2.31 μV confirmed the absence of severe hypoxic injury, the specificity of this threshold for a good outcome was 96.5%. Through a case review of 5 patients with a poor outcome who showed an amplitude above 2.31 μV, we found that one patient temporarily recovered consciousness after rewarming, but this patient died of multiple organ failure. Two patients showed status epilepticus (SE) on continuous electroencephalography immediately before the SSEPs were recorded. Their DWI scans indicated diffuse hypoxic injury at 24 and 8 h after the SSEPs were recorded, and these patients ultimately died. The remaining 2 patients with a CPC score of 3 showed diffuse injury on DWI, and one of them showed SE before the SSEPs were recorded. In conclusion, the majority (3/4) of patients with an N20–P25 amplitude above the upper threshold but with severe hypoxic injury showed SE. Although many previous studies have described giant evoked potentials coincident with epileptiform discharges [38–41], the SSEP amplitudes in those cases were considerably larger than the amplitudes in our cases. Therefore, we are not sure whether epileptiform discharges serve as a confounding factor for the N20–P25 amplitude or produce additional neuronal injury.

The amplitude and morphology of N20 and P25 depend on the electrode location and recording time [36, 42]; however, we analyzed only the amplitudes of short-latency waves to determine whether N20 or P25 was present. Serial and multichannel cortical recordings would facilitate the determination of the exact amplitude threshold through further study. In addition, further analysis to determine the morphology of the meaningful SSEP wave might be needed. Therefore, we believe that our lower amplitude threshold should not be recommended as a guideline for WLST.

Amorim et al. recently proposed that WLST and a self-fulfilling prophecy considerably impact the validity of using SSEPs for the prediction of a poor outcome [43]. As cases of absent SSEPs in patients with a good neurological outcome have been reported, WLST is a potential confounder in many studies. They reported that the FPR for an absent N20 in predicting a poor neurological outcome, adjusted for a WLST rate of 80%, is substantially higher than generally believed. However, it remains unclear whether our results present some evidence against their hypothesis. First, although WLST is prohibited by law in South Korea, the treating physicians were not blinded to the prognosis, and DNR orders were accepted. Finally, among in-hospital deaths, the DNR permission rate was 37.2% (29 patients), and the mode of 15 deaths was the physician’s decision not to escalate therapy according to the family’s demand. Second, SSEP interpretation may be affected by various factors [13], and the interrater reliability of SSEP testing is limited, especially for patterns predicting a poor outcome [8, 13, 43]. In the present study, 24 of 216 recordings (11.1%) were judged to be unsuitable due to a noise level too high to allow reliable interpretation or absence of the N13 peak and were excluded from the analysis. Thus, using SSEPs in isolation to predict a poor outcome is unwarranted, and SSEPs should only be used as one test of many in a multimodal approach to predict the outcome and decide on WLST, as recommended by current guidelines [1]. However, amplitudes above the higher thresholds can be used to suggest further or advanced treatment.

Our study should be interpreted considering the following limitations. First, this study was performed in a single hospital and was a retrospective, registry-based study, which may decrease the generalizability of the results, including the amplitude thresholds. The test machine parameters and recording protocols, such as the filter bandwidth and the stimulus intensity, might variably alter the amplitude of cortical SSEPs [44]. Second, as mentioned above, the SSEP results were not blinded, which could potentially have influenced decisions regarding withholding advanced treatment. Third, because the SSEPs were recorded for clinical purposes, they were not obtained from patients who were awakened immediately after TH. This profound selection bias may limit any interpretation of these data for predicting a good outcome. The recording times were also inconsistent. Although we included these cases based on claims in the recent literature, mild TH does not have a significant impact on the amplitude or presence of SSEPs [45, 46], and a quarter of the SSEPs were recorded before rewarming. Therefore, a prospectively designed multicenter study is needed to increase the generalizability of our results.

## Conclusions

In this retrospective, single-center registry-based study, the absence of P25, especially the absence of the N20–P25 component, improves the sensitivity for predicting a poor outcome without jeopardizing the specificity. On the other hand, amplitude analysis was suitable for detailed prediction. The N20–P25 amplitude threshold for predicting a poor outcome (< 0.64 μV) had a prognostic value similar to that observed for pattern analysis, and an amplitude > 2.31 μV provided a sensitivity and specificity for predicting a good outcome of 52.9% and 96.5%, respectively. However, because of some limitations, results should be viewed with caution regarding the possibility of false positives and interpreted in a multimodal approach. A further larger multicenter prospective study is needed to increase the generalizability of our results.

## Additional files

Additional file 1: **Figure S1.** Patterns of diffusion-weighted imaging. (DOCX 923 kb)

Additional file 2: **Figure S2.** The receiver operating characteristic curves for Cerebral Performance Category scores 3–5 at 6 months showing the predictive powers of various prognostic tests and combination models in the subgroup that had all 3 prognostic tests (*n* = 114; CPC 1–2, 33; and CPC 3–5, 81). (DOCX 123 kb)

Additional file 3: **Table S1.** Area under the curve of various prognostic tests and combination models for Cerebral Performance Category scores 3–5 at 6 months in the subgroup that had all 3 prognostic tests (*n* = 114). (DOCX 18 kb)

## Data Availability

All data generated or analyzed during this study are included in this published article.

## Abbreviations

AUC: Area under the curve
CI: Confidence interval
CPC: Cerebral performance category
DNR: Do not resuscitate
DWI: Diffusion-weighted imaging
EEG: Electroencephalography
FPR: False-positive rate
IQR: Interquartile range
NSE: Neuron-specific enolase
ROC: Receiver operating characteristic
ROSC: Return of spontaneous circulation
SE: Status epilepticus
SSEP: Somatosensory evoked potential
TH: Therapeutic hypothermia
WLST: Withdrawal of life-sustaining treatment
