# Supplementary material for: Beyond dichotomy: patterns and amplitudes of SSEPs and neurological outcomes after cardiac arrest
Source: Crit Care. 2019 Jun 18;23:224. doi: 10.1186/s13054-019-2510-x (PMC6582536; doi:10.1186/s13054-019-2510-x)
Supplement: Supplementary file 1 — Figure S1. Patterns of diffusion-weighted imaging. (DOCX 923 kb) [file 13054_2019_2510_MOESM1_ESM.docx]

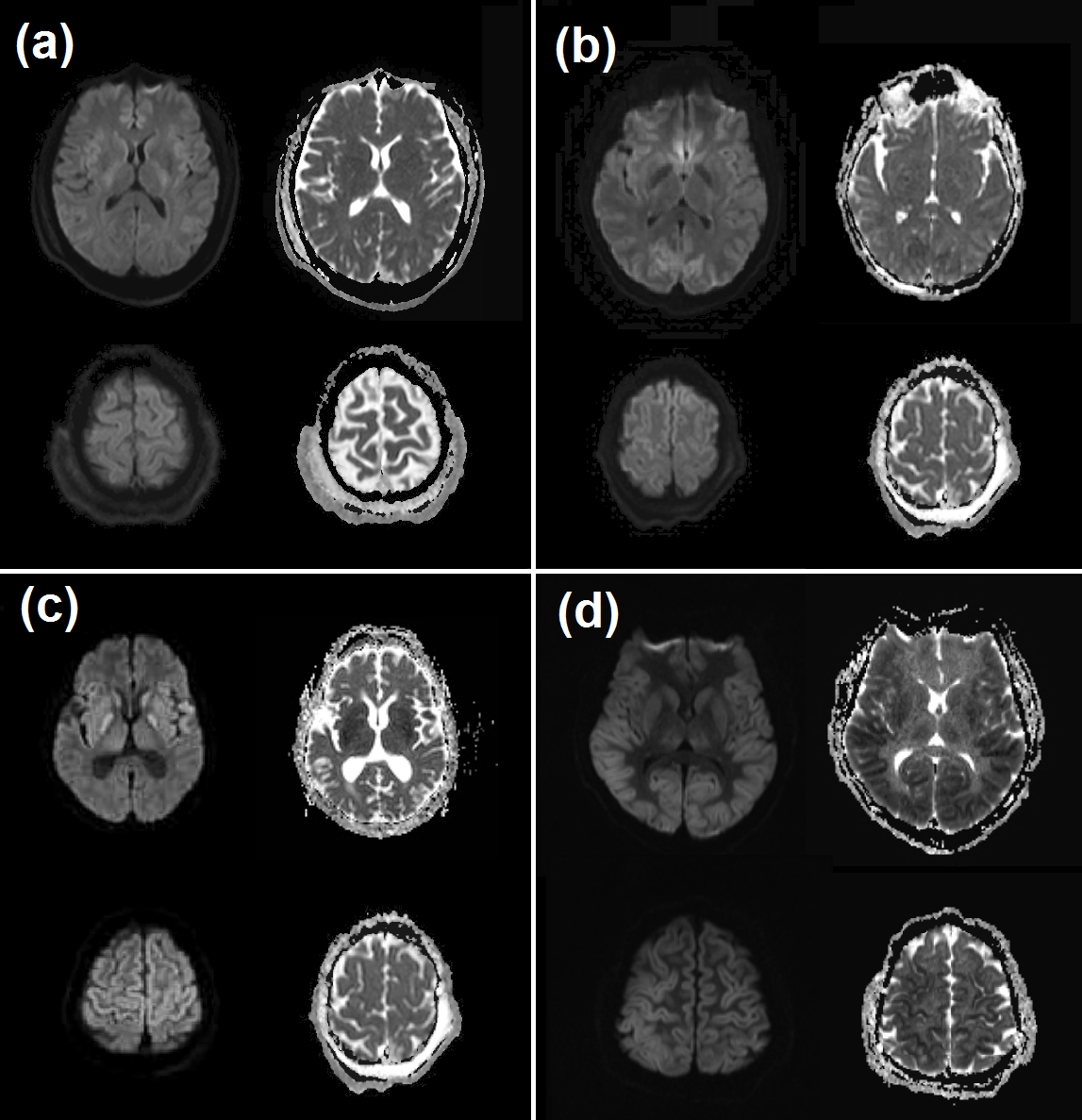


Figure S1. Patterns of diffusion-weighted imaging.

DWI findings were categorized into four patterns on the basis of the diffusion-restriction (hyperintense on DWI and low ADC values) lesions of the brain: (1) no diffusion-restriction lesion (figures a), (2) diffusion-restriction lesions, isolated cerebral cortex or deep grey matter (figure b), (3) multifocal lesions of diffusion-restriction, including both cerebral cortices and deep grey matter (figure c), and (4) global diffusion-restriction lesions in the brain (figure d).

DWI, diffusion-weighted imaging; ADC, apparent diffusion coefficient.
