# Supplementary material for: Beyond dichotomy: patterns and amplitudes of SSEPs and neurological outcomes after cardiac arrest
Source: Crit Care. 2019 Jun 18;23:224. doi: 10.1186/s13054-019-2510-x (PMC6582536; doi:10.1186/s13054-019-2510-x)
Supplement: Supplementary file 2 — Figure S2. The receiver operating characteristic curves for Cerebral Performance Category scores 3–5 at 6 months showing the predictive powers of various prognostic tests and combination models in the subgroup that had all 3 prognostic tests (n = 114; CPC 1–2, 33; and CPC 3–5, 81). (DOCX 123 kb) [file 13054_2019_2510_MOESM2_ESM.docx]

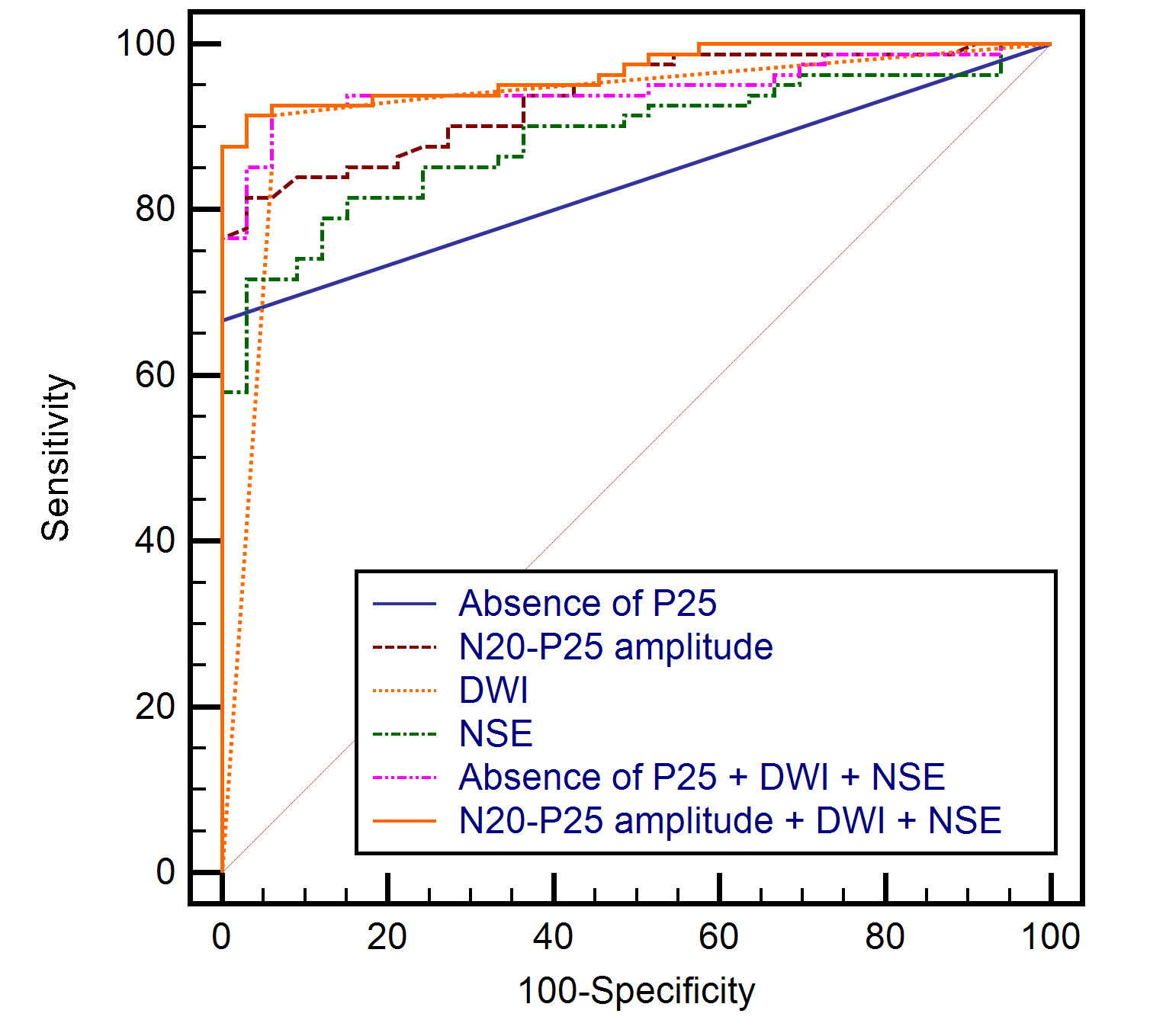


Figure S2. The receiver operating characteristic curves for Cerebral Performance Category scores 3–5 at 6 months showing the predictive powers of various prognostic tests and combination models in the subgroup that had all 3 prognostic tests (n= 114; CPC 1–2, 33; and CPC 3–5, 81).

SSEP, somatosensory evoked potentials; DWI, diffusion-weighted imaging; NSE, neuron-specific enolase; CPC, Cerebral Performance Category.
