# Supplementary material for: Beyond dichotomy: patterns and amplitudes of SSEPs and neurological outcomes after cardiac arrest
Source: Crit Care. 2019 Jun 18;23:224. doi: 10.1186/s13054-019-2510-x (PMC6582536; doi:10.1186/s13054-019-2510-x)
Supplement: Supplementary file 3 — Table S1. Area under the curve of various prognostic tests and combination models for Cerebral Performance Category scores 3–5 at 6 months in the subgroup that had all 3 prognostic tests (n = 114). (DOCX 18 kb) [file 13054_2019_2510_MOESM3_ESM.docx]

Table S1. Area under the curve of various prognostic tests and combination models for Cerebral Performance Category scores 3–5 at 6 months in the subgroup that had all 3 prognostic tests (n=114).

|  | AUC | 95% CI |
| --- | --- | --- |
| Absence of P25 | 0.83 | 0.75–0.90 |
| N20-P25 amplitude | 0.94 | 0.87–0.97 |
| DWI | 0.93 | 0.86–0.97 |
| NSE | 0.89 | 0.81–0.94 |
| Absence of P25 + DWI + NSE | 0.95* | 0.89–0.98 |
| N20-P25 amplitude + DWI + NSE | 0.97** | 0.92–0.99 |

*AUC of combination model including the absence of P25 is signifiantly different with those of the absence of P25 and the peak level of NSE (respectively, p<0.001 and p=0.006).

**AUC of combination model including the N20-P25 amplitude is signifinatly different with those of the N20-P25 amplitude and the peak level of NSE (respectivley, p=0.04 and p=0.002).

SSEP, somatosensory evoked potentials; DWI, diffusion-weighted imaging; NSE, neuron-specific enolase; AUC, area under the curve; CI, confidence interval.
